# Blood in the Urine—Some Cases

**Published:** 1909-03

**Authors:** Alfred L. Fowler

**Affiliations:** Atlanta, Ga.; Professor of Genito-Urinary Surgery and Venereal Diseases in the Atlanta College of Physicians And Surgeons; Genito-Urinary Surgeon to the Grady Hospital; Physician and Surgeon to the United States Penitentiary Hospital; Surgeon to St. Joseph’s Infirmary, Etc.


### BLOOD IN THE URINE—SOME CASES.*

(Continued).

BY ALFRED L. FOWLER, M. D., ATLANTA, GA.

PROFESSOR OF GENITO-URINARY SURGERY AND VENEREAL DISEASES IN THE ATLANTA COLLEGE OF PHYSICIANS AND SURGEONS; GENITO-URINARY SURGEON TO THE GRADY HOSPITAL; PHYSICIAN AND SURGEON TO THE UNITED STATES PENITENTIARY HOSPITAL; SURGEON TO ST. JOSEPH'S INFIRMARY, ETC.

pi and papillomata if they be present. If these are the cause of the hemorrhage they may be removed with the currette or the galvano-cautery.

3.—The next step in exploration of the bladder. With a velvet-eyed, soft rubber catheter we obtain urine directly from

*Read before the Fulton County Society of Medicine, January 7, 1909.

the bladder which should be kept and examined microscopically. The bladder is then irrigated with a 1 to 4,000 oxycyanat of mercury solution, followed by an injection of a dram of 4 per cent. cocaine solution with an Ultzman syringe into the prostatic and membranous urethra. General anesthesia has its disadvantages and is seldom necessary. After waiting about three minutes we may introduce the cystoscope; and it is advisable to employ both the prismatic and the direct view instruments. The former is more comfortable to the patient and, besides giving a better view of the ureters and trigonal tissue, prostatic hypertrophy and bleeding can often be seen. In order to see the prostate and anterior wall of the bladder, it is sometimes necessary to employ the retrograde cystoscope.

4.—Cystoscopy. For catherizing the ureters and obtaining a better view of the fundus, I prefer, and generally employ Cabots direct view double-barrelled irrigating cystoscope.

Bladder dilatation by water excels by far the method of air dilatation; and in reason, because in the former we have a cool medium; moreover, an antiseptic medium; and then, too, the bladder is accustomed naturally, to containing a fluid, while air, now and then, acts upon it like a foreign body. Notwithstanding the disadvantages of air dilatation it serves our purpose when making local applications to a diseased area, if it be localized. When it is dilated by a clear, antiseptic fluid (e. g., oxycyanat of mercury 1 to 4,000 or 5,000) its folds are smoothed out and villious tumors, if present, may be easily detected floating free in the liquid medium. Also, if jets of blood or pus are issued from the ureters, if villous or papillomatous processes are bleeding, or if the prostate is causing the hemorrhage, or a vesical calculus, or ulcers be present, the cystoscope will bring them clearly into view.

Failure to observe a stone occurs in difficult cases where the calculus is embedded in a sacculation. Likewise, failure to locate the ureters occurs when they lie between folds of mucous membrane or are covered over by trabeculae. But such cases are the exception and generally it can be determined with certainty which kidney is affected and also the functionating power of each kidney. This functionating power may be determined by injecting methylin blue or phloridzin subcutaneously and noting the time that the urine becomes discolored, collecting it separately with

*ureteral* catheters. Albarran produces polyuria, experimentally, with a diurectic mineral water and collects the urine every half hour. The amount, its molecular concentration and amount of urea and chlorides are determined in percentages. His findings have been that in normal kidneys, the molecular concentration and the percentage of urea and chlorides diminish in the same proportion as the amount of urine increases. In diseased kidneys he usually found that the amounts of urea and chlorides diminished in disproportion to the increase in the amount of fluid excreted. *Cryoscopy* is of much value in this connection.

Cystoscopy has a distinct place, apart from its service as a means of diagnosis. It enables us, guided by the eye, to remove favorably located tumors with the snare, also to crush stones when not too large, with the cystoscopic lithotrite, and further to lavage the pelvis of the kidney in pyelitis and subsequently cure it.

Henry Meyer, of San Francisco, has recently reported some brilliant intra-vesical operations without the knife and Lewis Wine Bremerman, of Chicago, has lately been doing excellent work with the cystoscopic lithotrite, as devised by Walker, of Baltimore. In many cases cystoscopy reveals the kind of tumor we have to deal with as well as the location of the growth.

Contra-indications: Any acute inflammation of the bladder contra-indicates cystoscopy; and in such conditions it is not only harmful, but difficult. This is likewise true of the urethra and urethroscopy. In tuberculosis of the bladder it seems to aggrevate the condition even more than other measures; and much harm can be done by unskillful cystoscopy in cases of villous tumors, particularly.

### TREATMENT.

The proper treatment of hematuria involves not only the removal of the cause, when it can be determined, but also the associated conditions that not infrequently go hand-in-hand with it.

In inflammation of the bladder and kidneys, hot sitz baths and hot fomentations, together with saline cathartics, urinary antiseptics and diuretics are indicated. The diet (the diet should consist chiefly of bread and milk. The latter, while not alkaline primarily, shortly becomes so in reaction. Asparagus, tomatoes, strawberries, etc., on account of the oxalic acid and a more complex substance called mercapton, which they contain, should be carefully avoided, as they act as irritants to the uro-genital membrane) should be as largely non-nitrogenous as possible, with as much absolute rest as can be obtained.

If the cause is attributed to the prostate, hot sitz baths, and the psychophore per rectum, as hot as the patient can comfortably stand it, are palliative measures not to be overlooked; also laxatives for the relief of venous obstruction, and removal of urethral constractures either by operation or dilation, according to the cause and location.

In acute inflammatory conditions no attempt should be made, as a rule, to check the hemorrhage. Oil of turpentine, ergot, gallic acid, and other astringents, when administered by the mouth are of no value in *vesical* hemorrhage. In persistent hemorrhage of the anterior urethra lasting for three days, I once used locally a 15 per cent. Monsels solution very effectively. In slight vesical hemorrhage my experience has taught that aluminum sulphate, 2 to 10 per cent., and silver nitrate, from 1 to 1,000-500, used as hot irrigations (115-120 degrees F.) are the most reliable. Solutions of suprarenal extract frequently not only fail to do good, but they are irritating and occasionally start up a "sledge-hammer" pulse which, of itself, may bring about a condition equal to, or exceeding in gravity, the vesical hemorrhage. I have observed this particularly in bladders that have lost their tone.

Dr. Paul Sauvan, of Montpellier, France, is an earnest advocate of antipyrine in all forms of diffuse bleeding. He states that the vaso-constrictive action of this drug is not followed by paralytic dilatation, and recommends a 5 to 10 per cent. solution. Clinically, he finds that more than 150 grains have to be absorbed to produce toxic accidents. Administered internally antipyrine has no hemostatic action.

Vesical hemorrhage, the result of stone, is easily handled and preferably by suprapubic cystotomy. Villous or papillomatous tumors when located favorably may be removed by the snare. These tumors, now and then, give rise to desperate hemorrhage and such a condition calls loudly for suprapubic cystotomy. Nothing is gained in these cases by delay. The bladder should be opened, the tumor snipped off, and the actual cautery applied to the bleeding point, if necessary. A 5 candle power incandescent light with which to illuminate the bladder facilitates the operation. The bladder wall stands the actual cautery very well, a fact that is also well attested in Botini's operation for hypertrophied prostate.

Malignant growths occasioning vesical hemorrhage of the bladder demand a suprapubic cystotomy. The tumor should be excised so as to include a portion of the healthy bladder wall. If this principle is not adhered to we are practically inviting its return.

Tuberculosis of the bladder and tubercular ulcers giving rise to vesical hemorrhage, can only be controlled by removing the cause.

If of the descending type nephrectomy will not only stay the process, but will be followed by a complete cure, once the primary tuberculous focus is removed. Irrigations of bichloride of mercury, beginning with a 1 to 10,000 or 1 to 5,000 solution about twice a week and gradually increasing to 1 to 1,000 and 1 to 500, probably gives the best results, locally. In cases of long standing the bladder is usually contracted and irrigations are not well tolerated.

I have seen a 20 or 30 per cent. solution of argyrol when injected into the bladder act almost like magic in allaying the pain and tenesmus in these cases, but have never observed any permanent results following its use. The usual medical (dietetic and hygienic) remedies may be tried; occasionally they seem to exert a favorable influence.

Tumors of the kidney and its pelvis, if they are the underlying cause of the hemorrhage, should be handled in the most radical surgical manner. Treatment consists in complete extirpation of the offending kidney, with as much ureter as possible, because these tumors frequently invade the ureters. Surgical interference depends entirely upon the diagnosis of disease in one gland only. By reason of our modern instrumentation and technique this can very readily be ascertained.

For stone in the kidney either nephrolithotomy or nephrotomy, dependent upon suppuration being present, is the operation.

Before reporting a few cases of hematuria, I desire to impress upon you these points:

1. The danger of acting too quickly before studying the case carefully.

2. The great danger in delaying in desperate hemorrhage.

3. The danger of aggravating the hemorrhage by clumsy instrumentation, such as cystoscopy, even when practiced by the experienced.

4. The preparatory treatment of the patient prior to instrumentation, particularly cystoscopy.

5. The value of local anesthesia in suprapubic cystotomy, particularly when the patient's condition contra-indicates general anesthesia.

6. The occasional occurrence of hematuria the result of acutely inflamed hemorrhoids.

CASES.

1. Hemophilia. Mr. R. L. D., age 28, nativity, Louisiana, occupation, teamster. Patient asserted that he had gonorrhoea seven years ago. Diagnosis: Organic urethral stricture at peno-scrotal junction, and varicocele. Patient applied March 17, 1906, to seek relief for his stricture, he being unaware of his varicocele. Examination disclosed a dense, fibrous, inelastic stricture at the peno-scrotal junction. Internal urethrotomy was advised, to which the patient consented. After cutting this stricture a persistent urethral hemorrhage followed, which continued three days and nights. My suspicions were shortly aroused and, upon questioning him, I learned, that hemophilia existed in his family; and that on a former occasion he nearly bled to death as a result of an accidental fall, when he cut his lips with his teeth. Hot compresses, the usual styptics, and adrenalin gave no relief. On the third night the loss of blood continued unabated and the patient's vitality had begun to wane and little hope was entertained for his recovery, it being estimated that he had passed a pint of blood within the last three hours. Everything else having been tried, a 15 per cent. Monsel's solution was injected with an Ultzman's syringe into the region of the stricture; the hemorrhage ceased immediately and a long bloodclot about the size and shape of a pencil was expelled a few hours later, the patient making an uneventful recovery.

2. Hemorrhoids. Mr. W. C., age 32; nativity, North Carolina; occupation, soldier. Patient presented himself because he said he was passing blood in his urine; had noticed it for ten days past, but never before. Urethral examination; negative. No veneral history. Rectal examination; showed acutely inflamed hemorrhoids. The prostate was found to be distinctly enlarged, soft and tender. Several specimens of urine obtained at various times showing a varying amount of hemorrhage, and occasionally clots. Centrifugalized urine revealed red blood cells, caudate cells and a few calcium oxalate crystals. Microscopic examination otherwise negative. The first and second glass tests were always clear. No symptoms of cystitis. Patient was sent to hospital and put to bed until acute inflammation of hemorrhoids had subsided. Meanwhile, the bladder was irrigated with potassium permanganate, boric acid, and silver nitrate solution. Urinary antiseptics and saline cathartics were administered with no appreciable effect. On September 18, 1906, assisted by Dr. A. P. Flowers, I operated on this patient for hemorrhoids. A good recovery. Two days afterwards the hematuria disappeared; and two weeks after this, examination of prostate showed that it had diminished in size and that its tenderness had disappeared. At last account, six months ago, there had been no return of hematuria. This case illustrates very clearly that hemorrhoids are, though rarely, a causative factor in vesical hemorrhage. When we consider the intimate association of the hemorrhoidal and prostatic plexuses in their relation to the neck of the bladder, the prostate and the anus, the case is not so surprising after all.

3. Villous Papilloma. Mr. J. M., age 41; nativity, New York; occupation, electrician. Patient appeared January 3, 1907, for relief of urinary hemorrhage. First glass test, clear; second and third glass tests showed a moderate amount of hemorrhage. Patient himself called my attention to what he called "a few small particles of fatty substances" mixed with the blood and urine. No cystitis present; and patient appeared to suffer more mentally than physically. Centrifugalized urine showed an abundance of red blood cells together with squamous epithelium and a few uric acid crystals. A diagnosis of villous tumor was made. Patient was put to bed; milk and bread diet ordered, together with cystogen as an urinary antiseptic. He was irrigated twice weekly with a 2 per cent. solution of aluminum sulphate, which was gradually increased in strength to 15 per cent. solution. After the third week both the hemorrhage and villous particles began to disappear and by the fourth week there were no further evidences of either. Eight months after there had been no return and cystoscopy showed a practically normal bladder with no tumor present.

4. Passive Congestion of Kidney. Mr. B. F. H., age, 26; nativity, Georgia; occupation, painter. Early in March patient consulted me because he said he was urinating blood. He admitted having had syphilis four years previously. Physical examination showed a hypertrophied heart with a distinct mitral and aortic regurgitation. Specimens of urine passed in my presence and also specimens brought for examination showed the urine to contain an uniformly diffused hemorrhage of a dark color. Examination of the urethra, bladder and prostate proved negative. Kidneys could not be palpated. Diagnosis: Passive congestion of the kidney, due to obstructive cardiac lesion. Patient was ordered to have inunctions of mercury, and protoiodide of mercury internally. This had a happy effect as long as the treatment was carried out; but the patient was indifferent and as soon as the hematuria began to clear he would discontinue treatment. Patient passed from under my observation eight months ago.

5. Active Hyperaemia of Prostate. History similar to vesical calculus. Case referred by Dr. J. M. Goldsmith. Mr. A. F. McD., age 19; nativity, Florida; occupation, clerk. Father died of acute Bright's at 49. No venereal history. Began to notice blood clots in urine one year ago. Activity increased the number of urinations. Seldom felt the urgency of urination at night. Occasional backache. Once or twice he noted urination was suddenly arrested. Had pain in region of perineum now and then. Pain always followed urination, lasting two or three minutes. Exercise increased pain; rest lessened it. Hemorrhage only present in third glass test. Residual urine ounces 1. Physical examination: Kidneys could not be palpated; pain absent over bladder; no hemorrhoids. Had previously been treated for cystitis through error of diagnosis. Was examined with stone searcher, but no stone present. Palpation of prostate per rectum, showed a moderately enlarged and tender prostate. Cystoscopy disclosed a large, bulging prostate, acutely congested, also thick mucus. Ureteral openings: Normal. Prostate was massaged and expression contents examined, which were largely composed of thick, viscid mucus and red blood cells. Treatment: Gentle massage of prostate twice weekly, alternating with full size Guyon sounds, and bladder irrigated with nitrate of silver, 1 to 4,000, gradually increasing to 1 to 500. All symptoms subsided in three weeks. Has had no treatment for past four months; neither has there been any return of symptoms.

6. Pedunculated Papilloma of Prostatic Urethra. Mr. J. D. B., age, 30. Case referred to our genito-urinary clinic by Dr. E. M. McDonald, November 23, 1908. Patient began to notice pain in lower lumbar region 3 years ago, which was followed by frequent micturitions, 12 to 15 times daily. Two and a half months ago he began to pass a slight amount of blood with first flow of urine. Palpation of prostate showed it slightly enlarged and tender with a few nodules. No history of gonorrhoea. No median bar. Examination of prostatic urethra with a Goldschmidt's irrigating posterior urethroscope disclosed, to Dr. Bryant and myself, a pedunculated papilloma about the size of a pea, situated just to the left of the verumontanum. I photographed this papilloma with a photographing urethroscope and hope to show you the negative later. Patient is to return shortly when we propose removing the tumor with a snare or the actual cautery.

NOTES.

Presumptive evidence of:

1. Vesical or renal hemorrhage.

2. Tumors of kidney.

3. Rest amounts to nothing in renal hemorrhage.

4. Neck of bladder, hypertrophied prostate and vesical calculus.

5. Renal.*

6. Prostatic.

7. Villous tumors of the bladder. Solution of adrenalin, aluminum sulphate, etc., when introduced into the bladder will not check renal hemorrhage.

8. Urethra.

9. Neuroses of bladder.

10. Vesical calculus and prostatitis.

11. Posterior urethritis, or cystitis.

12. The bladder capacity remaining intact, this is presumptive evidence of diabetes mellitus, diabetes insipidus, chronic interstitial nephritis, and urina spastica.

13. Patients with stone, as a rule, sleep all night without having to void their urine.

14. Hypertrophied prostate of chronic prostatitis.

15. The pain of renal colic occurs on the side corresponding to the diseased kidney and radiates along the ureter down into the groin or testicle.

16. Spasmodic pains and affections of bladder.

17. Urethral stricture—pain at sight of obstruction.

Bibliography: Ayers, Cabot, Casper, Christian, Erichsen and Lydston.

928-929 Candler Building.

*The rule that "the darker and more diffuse the blood is, the more renal source." Fenwick states, should absolutely be rejected.

